# Vaginal and Uterine Bacterial Communities in Postpartum Lactating Cows

**DOI:** 10.3389/fmicb.2017.01047

**Published:** 2017-06-08

**Authors:** Brooke A. Clemmons, Sydney T. Reese, Felipe G. Dantas, Gessica A. Franco, Timothy P. L. Smith, Olusoji I. Adeyosoye, Ky G. Pohler, Phillip R. Myer

**Affiliations:** ^1^University of Tennessee Institute of Agriculture, University of Tennessee, KnoxvilleTN, United States; ^2^U.S. Meat Animal Research Center, Agricultural Research Service, United States Department of Agriculture, Clay Center, NEUnited States; ^3^Department of Animal Sciences, Obafemi Awolowo UniversityIle-Ife, Nigeria

**Keywords:** uterine, vaginal, bacteriome, cow, reproductive success

## Abstract

Reproductive inefficiency in cattle has major impacts on overall productivity of cattle operations, increasing cost of production, and impacting the sustainability of the cattle enterprise. Decreased reproductive success and associated disease states have been correlated with the presence of specific microbes and microbial community profiles, yet details of the relationship between microbial communities and host physiology are not well known. The present study profiles and compares the microbial communities in the bovine uterus and vagina using 16S rRNA sequencing of the V1–V3 hypervariable region at the time of artificial insemination. Significant differences (*p* < 0.05) between the vaginal and uterine communities were observed at the level of α-diversity metrics, including Chao1, Shannon’s Diversity Index, and observed OTU. Greater clustering of vaginal OTU was apparent in principal coordinate analysis compared to uterine OTU, despite greater diversity in the vaginal community in both weighted and unweighted UniFrac distance matrices (*p* < 0.05). There was a significantly greater relative abundance of unassigned taxa in the uterus (*p* = 0.008), otherwise there were few differences between the overall community profiles. Both vaginal and uterine communities were dominated by Firmicutes, although the relative abundance of rRNA sequences corresponding to species in this phylum was significantly (*p* = 0.007) lower in the uterine community. Additional differences were observed at the genus level, specifically in abundances within *Clostridium* (*p* = 0.009), *Anaerofustis* (*p* = 0.018), *Atopobium* (*p* = 0.035), *Oscillospira* (*p* = 0.035), *5-7N15* (*p* = 0.035), *Mycoplasma* (*p* = 0.035), *Odoribacter* (*p* = 0.042), and within the families *Clostridiaceae* (*p* = 0.006), *Alcaligenaceae* (*p* = 0.021), and *Ruminococcaceae* (*p* = 0.021). Overall, the comparison revealed differences and commonalities among bovine reproductive organs, which may be influenced by host physiology. The increased abundance of unassigned taxa found in the uterus may play a significant biological role in the reproductive status of the animal. The study represents an initial dataset for comparing bacterial communities prior to establishment of pregnancy.

## Introduction

Reproductive efficiency is necessary for the survival of any species. Failure to reproduce or maintain pregnancy represents a significant cost to many species, including humans, exotic or endangered species, and domestic livestock. One *in vitro* fertilization cycle in humans averages ∼$12,000 (Thompson, 2016). In endangered or exotic species, breeding can often be a limiting factor, particularly in captivity, leading to possible extinction (Snyder et al., 1996). In domestic livestock, specifically cattle, reproductive loss and inefficiency results in ∼$600 million to $1.4 billion lost annually (Bellows et al., 2002; De Vries, 2006). While these losses are multifaceted and influenced by many factors such as disease states (Michi et al., 2016), male and female gametes (Druet et al., 2009; Valour et al., 2015), and genetics related to male and female fertility (Berry et al., 2014; Carthy et al., 2014), these factors cannot account for all reproductive related losses.

Human studies using next generation sequencing have revealed correlations between various reproductive issues in women, such as higher risk of preterm labor or fertility issues, and microbial factors, including presence of specific populations of microbes. One study found that lower abundances of *Lactobacillus* and increased bacterial diversity in the vaginas of pregnant women were associated with shorter gestational length (DiGiulio et al., 2015). The vaginal environment is an important aspect of the reproductive system because it represents the first obstacle to sperm, as well as the first barrier to protect the upper reproductive tract. Vaginal pH has been associated with different bacterial populations in humans, namely *Lactobacillus*, and is thought to aid in protection against infection (Boris and Barbés, 2000) providing evidence that the microbiota of a healthy woman may contribute significantly to the reproductive success and prevention of a diseased state. The study conducted by DiGiulio et al. (2015) concludes that such findings contribute toward a better understanding of the human body, possibly leading to novel methods for resolving or preventing reproductive issues or idiopathic infertility. At minimum, the presence or absence of specific microbial species might be useful indicators of pregnancy complications. Moreover, microbial biotechnology holds promise for combating some of these aforementioned reproductive issues (Huang et al., 2014; DiGiulio et al., 2015; Prince et al., 2016).

Culture-dependent methods examine only a small proportion of the overall microbial population (Amann et al., 1995; Wintzingerode et al., 1997), because many species of microbes, including those found in sensitive and unique microenvironments such as the uterus and vagina, cannot be cultured in a lab, possibly due to difficulty replicating their normal environment (Amann et al., 1995; Hugenholtz and Pace, 1996; Zoetendal et al., 2004). However, recent projects, based on next generation sequencing, such as the Human Microbiome Project, have propelled microbiome–host interaction research forward within the past decade, and that knowledge base continues to increase (Schuster, 2007; Human Microbiome Project Consortium, 2012).

Reproductive inefficiency in cattle can be extremely costly to producers. In dairy herds, economic based models have suggested that an average single pregnancy at conception is valued at US $278, and pregnancies lost after day 30 will cost a producer upward of US $550 (De Vries, 2006). While researchers continue to scrutinize mechanisms and approaches to reduce these losses and advance reproductive health, little has progressed regarding the microbiota within the reproductive tract of female bovines (Santos and Bicalho, 2012; Swartz et al., 2014; Stevenson et al., 2015). In addition to few studies conducted characterizing the microbiota of vaginal communities, fewer yet have interrogated uterine microbial communities in cattle. This limited research has predominantly been conducted in dairy cattle, which can vary significantly from beef cattle physiologically (Hart et al., 1975; Hayes et al., 2009; Knudsen et al., 2012; Santos and Bicalho, 2012). Determining the host-microbe relationship within the reproductive tract will provide stakeholders with better tools for improving reproductive success.

A few studies have examined both the uterine and vaginal microbiomes in humans, but little data exists about these microbiomes in livestock species or their potential role in reproductive success of animals. The influence of estrous cycles and their effects on these microbiomes are of particular interest in bovine reproductive physiology. For this study, the use of standard synchronization protocols for artificial insemination (AI), involving injections of hormones to bring a group of animals into similar hormonal status, reduces possible confounding effects due to varying hormonal levels and physiological states. The present study explores the potential of this approach, by focusing on vaginal and uterine microbiomes of synchronized animals 2 days prior to AI. These data are used to assess the approach of bacterial community profiling in the context of similar hormonal statuses and the same period in the bovine reproductive cycle. Non-pregnant cows were chosen for this initial screening to remove any influence that pregnancy, including hormonal and physiological factors, might have on the vaginal and uterine bacterial communities present. Ultimately, this foundational information could form part of a more comprehensive approach profiling reproductive tract microbiomes across the full reproductive cycle, and potentially create novel diagnostic criteria to elucidate likelihood of reproductive success.

## Materials and Methods

### Experimental Design and Sampling

This study was carried out in accordance with the recommendations of the Institutional Animal Care and Use Committee at the University of Tennessee, Knoxville. The protocol was approved by the University of Tennessee, Knoxville Institutional Animal Care and Use Committee.

All cows involved in this study were part of the East Tennessee Research and Education Center (ETREC) registered Angus herd. Thirty cows were selected from the fall calving herd ranging from 3 to 11 years of age with an average of 5 years at initiation of the study. Cows were an average of 82 days postpartum at AI. Cows included in the study had daughters retained at ETREC for use in future longitudinal studies of the microbiome. All cows were kept on cool-season grass pastures supplemented with corn silage for the duration of the study.

All 30 cows were inseminated following an industry-standard 7-Day Co-Synch Protocol, with the addition of a pre-synchronization step to help establish baseline cyclicity in the herd. Controlled internal release device (CIDR) were not used due to collection methods. The pre-synchronization protocol was performed 21 days prior to AI (D-21), by intramuscular injection of 5 mL of 25 mg/mL prostaglandin. A standard Co-Synch Protocol was then begun at D-9 by injection of 2 mL of 100 mcg/mL gonadotropin releasing hormone, to initiate a follicular wave. The animals were given an administration of 5 mL of 25 mg/mL prostaglandin on D-2 to regress the corpus luteum, at the same time that flushes for microbiome profiling were collected. The uterine and vaginal flush with saline solution in all cows were performed as follows: syringe injection of 60 mL into the vagina, followed by rectal massage to collect the fluid (vaginal flush); inflation of a Foley catheter in the uterus after passing through the vagina, to sequester the uterus from the vagina, followed by introduction of 180 mL saline and rectal massage to collect the fluid (uterine wash). All wash samples were stored at -80°C until used for microbiome profiling. The animals then had injection of 2 mL of 100 mcg/mL gonadotropin releasing hormone on D0 to induce ovulation and AI was performed using semen from the same sire for all cows. Animals were subjected to pregnancy check at D30, and 10 non-pregnant (open) cows were identified. Non-pregnant animals were used for this study to eliminate any possibility of the flushing technique having an effect on the pregnancy establishment, thus biasing the results. The flushes from these 10 open animals were used to obtain total DNA for 16S rRNA profiling. Immediately after flush, pH of each sample was measured using UltraBasic pH meter (Denver Instruments, Arvada, CO, United States).

### DNA Extraction and Sequencing

Each flush sample was vortexed to homogenize, then a 5 mL aliquot was taken and centrifuged in 15 mL tubes at 4,696 ×*g* and 4°C, and pelleted material was resuspended in 180 μL of sterile saline solution to concentrate microbial cells. DNA was then extracted from the cell concentrate using the Qiagen DNEasy Blood and Tissue kit (Qiagen, Hilden, Germany) according to manufacturer instruction. Extractions were stored at -20°C until amplification. DNA was amplified using polymerase chain reaction (PCR) for 30 cycles at an annealing temperature of 58°C, targeting the V1–V3 hypervariable regions of the bacterial 16S rRNA gene. Modified universal primers 27F (5′-Adapter/Index/AGAGTTTGATCCTGGCTCAG) and 519R (5′-Adapter/Index/GTATTACCGCGGCTGCTG) including TruSeq indices and adapters were used with AccuPrime Taq high fidelity DNA Polymerase (Life Technologies, Carlsbad, CA, United States) to produce the sequencing libraries. Products were quality checked with gel electrophoresis. Libraries were then purified using AmPure beads (Agencourt, Beverly, MA, United States) and quantified using a Nanodrop 1000 spectrophotometer (Thermo Scientific, Wilmington, DE, United States) and by real-time PCR on the LightCycler 480 system (Roche Diagnostics, Mannheim, Germany). The PCR amplicon libraries were sequenced using the 2 × 300, v3 600-cycle kit and the Illumina MiSeq sequencing platform (Illumina, Inc., San Diego, CA, United States) at the United States Meat Animal Research Center (US MARC; Clay Center, NE, United States).

### Sequence Read Processing and Analysis

Sequence data is available from the NCBI Sequence Read Archive (SRA Accession SRP103314). Additional descriptive information is associated with NCBI BioProject PRJNA382146. Amplicon sequence reads were processed using the Quantitative Insights Into Microbial Ecology (QIIME) bioinformatics pipeline, version 1.9.1 (Caporaso et al., 2010) and Mothur version 1.36.1 (Schloss et al., 2009). Sequences were quality trimmed using the Galaxy server (Afgan et al., 2016) and those with a score ≥ Q25 were retained. Sequences that contained read lengths shorter than 300 bp were removed and adapters/index sequences were trimmed. Chimeric sequences were identified and filtered using usearch61 (Edgar, 2010). Sequences classified as chloroplasts and mitochondria were removed from the analysis. To avoid biases generated by differences in sequencing depth, each sample was subsampled to an even depth of 50,000 sequences. Operational taxonomic unit (OTU) picking was completed utilizing the cleaned subsamples and were clustered with a pairwise identity threshold of 97% using the UCLUST module from QIIME and further assigned to taxonomy using UCLUST and the Greengenes v13_8 16S rRNA database as a reference (Caporaso et al., 2010). Phylogenic trees were built with FastTree (Price et al., 2010) to determine α- and β-diversity metrics. Alpha-diversity was analyzed using observed species, Faith’s phylogenetic diversity, Shannon diversity, and Chao1 richness indices. Beta-diversity analyses were performed using weighted and unweighted UniFrac distance matrices, as implemented in QIIME. Principal coordinates analysis (PCoA) was performed using weighted and unweighted UniFrac analyses (Lozupone and Knight, 2005). Heatmaps were constructed utilizing OTU found within ≥50% of samples and at a relative abundance of ≥0.01%.

### Statistical Analysis

All variables were tested for normality using the PROC UNIVARIATE procedure in SAS 9.4 (SAS Institute, Cary, NC, United States). Differences in pH and bacterial community characteristics by normally distributed variables (number of observed OTU, and Chao1) were statistically analyzed by a one-way analysis of variance (ANOVA) for multiple independent groups (Weiss et al., 2017). Differences which do not follow a normal distribution and all multiple-group comparisons (Shannon’s index, Faith’s phylogenetic diversity and relative abundances of taxonomic profiles) were completed using the Kruskal–Wallis H test with Benjamini–Hochberg FDR multiple test correction (Weiss et al., 2017). Where appropriate, reported *p*-values are those corrected for multiple testing. Correlations were performed in SAS 9.4 using the PROC CORR procedure. Spearman’s correlation was used for all non-parametric data. For all analyses, the significance level was set at 0.05. Abundance values reported in the results and in the additional files are reported as mean relative abundances for the environment groups along with the standard error. Statistical differences in bacterial community composition between environments were calculated using analysis of similarity (ANOSIM) with 9999 permutations and principal coordinate analysis (PCoA) of weighted and unweighted UniFrac distances within QIIME.

## Results

### Uterine and Vaginal pH

All uterine and vaginal samples were measured for pH. Vaginal pH ranged from 6.15 to 7.44 with a mean of 6.69 ± 0.14 for individual vaginal samples. Uterine pH ranged from 5.62 to 6.52 with a mean of 6.06 ± 0.09. Individual uterine and vaginal pH data is available in Supplementary Material S1. Differences in pH did exist between reproductive environments (*p* = 0.0016), with a greater pH in the vagina than in the uterus. There was a weak negative correlation between the uterine and vaginal pH values (*r* = -0.2242; *p* = 0.5334).

### Sequencing Information

A total of 20 samples were processed for DNA, including vaginal and uterine samples from 10 non-pregnant animals at D-2 prior to AI. Open animals were chosen to establish native uterine and vaginal bacteriomes of cows undergoing timed AI. Bacterial communities were analyzed by amplifying and sequencing the V1–V3 hypervariable regions of the 16S rRNA gene. After quality control and chimera removal, a total of 2,080,553 remained among all 20 samples, for an average of 100,000 per sample (**Table 1**). Total number of cleaned sequences from uterine samples were 1,149,373 sequences and ranged from 97,855 sequences to 149,286 sequences for individual uterine samples. Total number of cleaned sequences from the vaginal samples were 931,180 sequences and ranged from 63,302 sequences to 125,486 sequences for individual vaginal samples. Average number of cleaned sequences with standard error are presented in **Table 1**.

**Table 1:** Sequence and alpha-diversity statistics of the 16S rRNA gene sequences for bacterial populations in the vaginal and uterine environments.

	Vaginal	Uterine
No. of cleaned sequences	93,541 ± 21,401	114,937 ± 16,774
Normalized no. of sequences	50,000	50,000
Observed OTU^1,2^	838 ± 144^a^	495 ± 208^b^
Chao1^2^	882 ± 130^a^	608 ± 201^b^
Faith’s Phylogenetic Diversity^3^	48.5 ± 2.30^a^	29.6 ± 4.38^b^
Shannon’s Diversity Index^3^	7.34 ± 0.69^a^	5.83 ± 1.05^b^


*^a,b^Within a row, means for the range of individual metrics having differing superscripts differ (*P* < 0.05).^1^ OTU, operational taxonomic units.^2^ Means between the two environments were compared using ANOVA.^3^ Means between the two environments were compared using Kruskal–Wallis H test.*

### Alpha- and Beta-Diversity

After binning reads at 97% similarity, a total of 13,328 OTU were detected, with an average of 838 and 495 OTU for the vaginal and uterine samples, respectively. Alpha-diversity was measured using observed OTU, Chao1, Faith’s Phylogenetic Diversity and Shannon’s Diversity Index, and is presented in **Table 1**. Significant differences existed in alpha-diversity between the uterine and vaginal bacterial observed OTU, Chao1, Faith’s Phylogenetic Diversity and Shannon’s Diversity Index (*p* < 0.05). The vagina had a significantly greater number of OTU than did the uterus, increased richness as measured by Chao1, and greater diversity as measured by Shannon’s Diversity Index and Faith’s Phylogenetic Diversity all of which are presented in **Table 1**. Core OTU are found in 100% of samples from each environment, and the shared core phylotypes are those core OTU present in both the uterus and vagina. The uterus contained 76 core OTU, the vagina had 279 core OTU, and of those core OTU, 36 OTU were shared between the environments.

**FIGURE 1:**
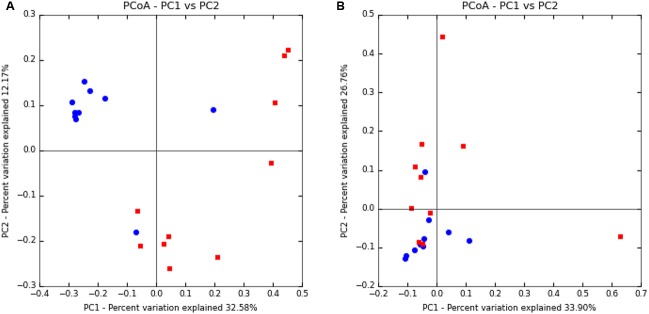
Principal coordinate analysis of uterine and vaginal samples using UniFrac unweighted **(A)** and weighted **(B)** metrics. Samples were analyzed from rarefied subsets of 50,000 sequences from each sample. Uterine samples (*n* = 10) are represented by red squares and vaginal samples (*n* = 10) are represented by blue circles.

Beta-diversity was also analyzed to examine differences in microbial communities between samples. Using an OTU-centric approach, PCoA matrices were employed using weighted and unweighted UniFrac distance matrices to compare the phylogenetic divergence among the OTU between vaginal and uterine samples (**Figure 1**). While vaginal samples contained a greater phylogenetic diversity and greater number of bacterial OTU, their spatial heterogeneity was reduced and thus tended to cluster more closely than the uterine samples in both the weighted and unweighted UniFrac distance matrices, where uterine samples had a greater spatial heterogeneity. Specifically, ANOSIM testing of the samples revealed that the compositions of the bacterial communities differed significantly (*p* = 0.005) between the uterus and vagina. The positive value of the *R* statistic (*R* = 0.1538) indicates clustering of samples based on the region interrogated, however, proximity of the value to zero supports the greater spatial heterogeneity of the samples observed in the PCoA. Overall, although the ANOSIM indicates significance, the *R* statistic dictates that the clustering is only moderate.

### Taxonomic Composition

**FIGURE 2:**
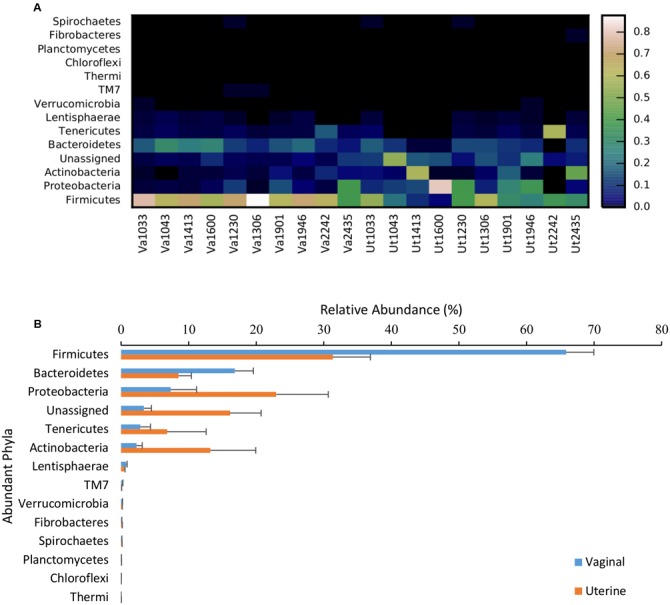
Relative abundance of bacterial phyla present in the uterus and vagina among samples (heatmap; **A**) and as a function of region (bar graph; **B**). Data consisted of phyla present in ≥0.01% relative abundance in ≥50% of the vaginal and uterine samples. Uterine (Ut) and Vaginal (Va) samples are denoted by animal ID.

In both the uterus and vagina, *Firmicutes* was the most dominant phyla with an average relative abundance of 31.3 ± 5.6% and 65.9 ± 4.1%, respectively, across all samples. In the uterus, the next most prevalent bacterial phyla based on relative abundance were *Proteobacteria* (22.9 ± 7.7%), *Actinobacteria* (13.2 ± 6.7%), and *Bacteroidetes* (8.5 ± 1.9%), with all other phyla present at <1% and unassigned taxa at 16.1 ± 4.6%. In the vagina, the other dominant phyla besides *Firmicutes* consisted of *Bacteroidetes* (16.8 ± 2.7%), *Proteobacteria* (7.4 ± 3.8%), *Tenericutes* (2.8 ± 1.5%), and *Actinobacteria* (2.3 ± 0.8%), with all other phyla representing <1% relative abundance and unassigned taxa at 3.4 ± 1.1%. The only significantly different phyla between the uterus and vagina were *Firmicutes*, which were greater in abundance in vaginal samples (*p* = 0.007), and unassigned taxa, which were reduced in abundance in vaginal samples (*p* = 0.008). A visual distribution of the phyla across cows and environment are available in **Figure 2**. Additional information containing phylum- and genus-level relative abundances as well as statistical information can be found in Supplementary Material S2.

**FIGURE 3:**
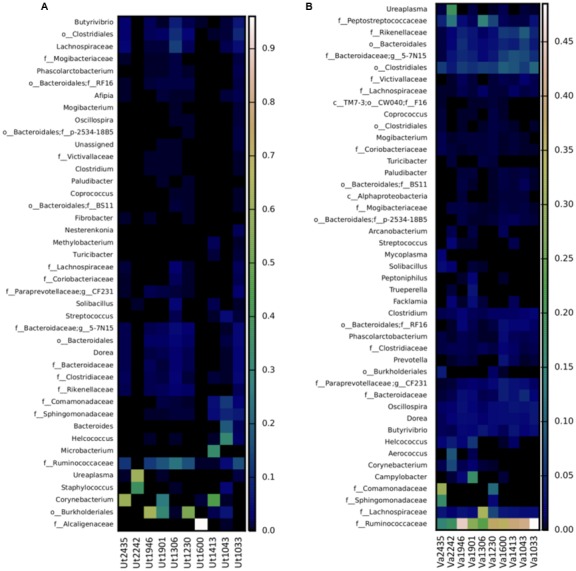
Heatmap of the taxonomic profile for bacterial genus-level relative abundance in uterine **(A)** and vaginal **(B)** samples. Data consisted of genera present in ≥0.01% relative abundance in ≥50% of the vaginal and uterine samples. Uterine (Ut) and Vaginal (Va) samples are denoted by animal ID.

At the genus level, the most prevalent genera in the uterus representing ≥1% relative abundance of the total sequences included *Corynebacterium* (10.6% ± 5.6), *Ureaplasma* (6.1% ± 5.9), *Staphylococcus* (3.7% ± 3.5), *Microbacterium* (1.6% ± 1.6), *Butyrivibrio* (1.4% ± 0.5), and *Helcococcus* (1.3% ± 1.0), which are represented in the heatmap in **Figure 3A**. The predominant genera in the vagina were an undetermined genus and family of the order *Bacteroidales* (3.4% ± 0.5), followed by genus *5-7N15* of family *Bacteroidaceae* (3.4% ± 0.6), and genera *Oscillospira* (2.0% ± 0.2), *Butyrivibrio* (1.8% ± 0.3), *Ureaplasma* (1.8% ± 1.6), *Campylobacter* (1.7% ± 1.4), *Dorea* (1.6% ± 0.2), *CF231* of family *Paraprevotellaceae* (1.5% ± 0.3), *Clostridium* (1.3% ± 0.1), *Helcococcus* (1.2% ± 0.5), and *Corynebacterium* (1.0% ± 0.6), as presented in **Figure 3B**. In contrast to analysis at the phylum level, where only *Firmicutes* were significantly different between vagina and uterus, several significantly different genera were identified between the two environments. At the genus level, the vagina contained significantly greater levels of undetermined genera of the family *Clostridiaceae* (*p* = 0.006), *Clostridium* (*p* = 0.009), *Anaerofustis* (*p* = 0.018), undetermined genera of family *Ruminococcaceae* (*p* = 0.021), *Atopobium* (*p* = 0.035), *Oscillospira* (*p* = 0.035), *5-7N15* of family *Bacteroidaceae* (*p* = 0.035), *Mycoplasma* (*p* = 0.035), and *Odoribacter* (*p* = 0.042). The uterus contained a greater relative abundance of undetermined genera of family *Alcaligenaceae* (*p* = 0.021).

The 16S rRNA sequences that could not be classified to the genus level across the uterine samples, included those in families *Ruminococcaceae* (10.89% ± 2.7), *Alcaligenaceae (8.1% ± 8.0)*, *Lachnospiraceae* (2.3% ± 0.8), *Sphingomonadaceae* (1.3% ± 0.5), *Comamonadaceae* (1.2% ± 0.6), *Bacteroidaceae* (1.2% ± 0.3), and *Rikenellaceae* (1.1% ± 0.3). In some samples, genera and families could not be identified, including orders *Burkholderiales* (9.6% ± 4.8), *Clostridiales* (3.5% ± 1.0), and *Bacteroidales* (2.0% ± 0.4). Vaginal samples that contained genera that could not be identified or placed into the undefined category included family *Ruminococcaceae* (32.6% ± 3.5), undetermined genera and family member of the order *Clostridiales* (7.1% ± 0.6), family *Lachnospiraceae* (5.7% ± 2.4), family *Peptostreptococcaceae* (3.7% ± 1.3), family *Rikenellaceae* (3.3% ± 0.7), family *Comamonadaceae* (2.8% ± 2.1), family *Sphingomonadaceae* (1.3% ± 1.1), family *Clostridiaceae* (1.2% ± 0.2), and family *Bacteroidaceae* (1.8% ± 0.4).

## Discussion

In humans and other animals, healthy microbial ecosystems within the body contribute to overall health and wellness of the host organism (Human Microbiome Project Consortium, 2012; Shreiner et al., 2015). While many studies have been conducted to identify bacterial and other microbial communities in the human vagina and uterus, few have interrogated these communities in cattle. Knowledge of microbiome influences in beef cattle stems primarily from studies conducted in the gastrointestinal tract, with little emphasis on the microbiome of the reproductive tract. This study aimed to conduct foundational research to interrogate the uterine and vaginal bacteriomes of cows undergoing estrus synchronization. Understanding bacterial communities in the reproductive tracts of cows will provide foundational data in order to ultimately determine how these factors are correlated with reproductive success. An increased comprehension of these communities may lead to novel approaches to improving reproduction, such as treatment with probiotics, to introduce microbial communities that result in positive results with regards to reproduction.

The most abundant bacterial phyla identified in the uterus in this study were, in order of abundance, *Firmicutes, Proteobacteria, Actinobacteria*, and *Bacteroidetes*, which corresponds with dominant phyla present in the vagina, with the exception of the presence of *Tenericutes* in vaginal communities. These phyla are representative of the most common phyla found in many environments, particularly in host-microbiome relationships. The uterine bacterial communities contained significantly greater unassigned or unidentified taxa than did the vaginal communities. Unassigned taxa accounted for 16.1% relative abundance in the uterus, compared to only 3.4% unassigned OTU in the vagina. The lack of existing studies of the uterine bacterial communities may indicate that many of these populations have yet to be determined, including their role in uterine physiology and possible correlations with reproductive health and success.

The common phyla found between the uterus and vagina are those found most commonly in many host-microbiome relationships in many species (Turnbaugh et al., 2009; Human Microbiome Project Consortium, 2012; Jami and Mizrahi, 2012; Myer et al., 2015); however, the ratios and relative abundances of these phyla are correlated with changes in host physiology. These common phyla have been shown to be independent of genetic lineage or sex (Ley et al., 2005). While the mechanisms explaining these relationships are not well characterized, there is a clear relationship between host phenotype and presence of different bacterial communities, abundances, and diversity in many species (Human Microbiome Project Consortium, 2012; Le Chatelier et al., 2013; Jami et al., 2014; Romero et al., 2014). Increased diversity in the microbiome has been associated with negative phenotypes in some species (Turnbaugh et al., 2009; Hyman et al., 2014; Durso et al., 2015). In contrast, stability of bacterial communities in the vagina have been associated with a healthy female reproductive tract (Gajer et al., 2012; Human Microbiome Project Consortium, 2012; DiGiulio et al., 2015).

Core OTU are those that are found across a habitat (Turnbaugh et al., 2007), and core OTU present a unique opportunity to identify those bacteria acting as keystone bacteria or functional groups that contribute to certain phenotypes in animals. While greater abundance of specific OTU does not necessarily indicate that those OTU perform necessary functions, it could provide insight into the health and maintenance of the animal. In mice and humans, core bacterial communities have been associated with phenotypic variation (Ley et al., 2005; Turnbaugh et al., 2009). Shared OTU between the uterus and vagina suggests that there is some interaction between the bacterial communities of the uterus and vagina. Yet, the core OTU differences between the uterus and vagina are likely a result of the functional differences associated with the tissues and microbial ecosystem niche.

Besides unassigned taxa, the uterus contained significantly greater abundances of unidentified genera in family *Alcaligenaceae* compared to the vagina. This family is predominantly aerobic, though some members of the family can use nitrate or nitrite as an electronic acceptor in lieu of oxygen (De Ley et al., 1986) since the uterus is predominantly an anaerobic environment. Heifers and cows can be grouped based on the level of aerobic bacterial communities in the vagina, regardless of age or pregnancy status (Laguardia-Nascimento et al., 2015). This suggests that variation among individuals, including anatomical and physiological differences, may influence the microbiome of the reproductive tract (Laguardia-Nascimento et al., 2015). Additional studies or analyses could be conducted to determine the impact of cow-specific differences on microbiome establishment.

Greater levels of diversity and increased richness in the vagina when compared with the uterus may be explained by its proximity to the external environment. The uterus is significantly more restricted from external exposure compared to the vagina (Sheldon et al., 2002). Protection of the uterine environment by the vagina and cervix may contribute to the decreased diversity present in the uterine cavity. The vaginal microbiome has been demonstrated to combat infectious bacteria, likely due to regulation of the vaginal pH (Boskey et al., 1999; Boris and Barbés, 2000; Brabin et al., 2005; van Oostrum et al., 2013; Huang et al., 2014). Maintaining a low vaginal pH prevents colonization of pathogenic microbes, which in turn can positively impact fertility (Boskey et al., 1999; Boris and Barbés, 2000; O’Hanlon et al., 2013).

Some bacteria may be responsible for maintaining the health of the vagina, which could in turn promote a healthy uterus. The relative abundances of OTU that were greater in the vagina than uterus are those that are abundant within the gastrointestinal tract or feces, particularly those in the genus *Clostridium*. The greater abundances of these bacteria in the vagina may be due to the anatomy of the cow’s reproductive tract but some mechanism likely reduces their presence in the uterus (Songer, 1996; Singh et al., 2008). Mechanisms may include a physical barrier provided by the cervix (Singer and Jordan, 2009; Nott et al., 2016); competition with other microbes more suited to the environment (Recine et al., 2016); antagonistic environment created by mutualistic microbes in coordination with the host (Klebanoff et al., 1991; Boskey et al., 2001; Kaewsrichan et al., 2006); or simply a constant introduction of these bacteria in the vagina from feces, providing constant opportunities for colonization.

Given the critical role that bacteria have in maintaining homeostasis within the uterine and vaginal environment (Mendez-Figueroa and Anderson, 2011), it is possible that microbiota associated with reproductive tracts may significantly affect the pregnancy rates and reproductive success in cows. Women with bacterial vaginosis (BV) have a greater incidence of infertility (van Oostrum et al., 2013). Cows and heifers that experience fertility or pregnancy maintenance issues may have different microbiota or proportions of core microbiota present in the reproductive tract. Additional research should aim to further analyze the microbiome of the uterus and vagina in cattle to determine relationships between presence (or absence) of certain microbiota and fertility rates. Such research would be utilized to document variation of the different abundances of microbiota in reproductive tracts of pregnant and non-pregnant cows, as well as identifying potentially unique microbes that affiliate with high versus low levels of fertility. It may be beneficial to also determine the microbiome changes associated with hormone shifts that occur throughout the estrous cycle, as these changes have been observed in humans (Human Microbiome Project Consortium, 2012). Determining the differences in bacterial and other microbial populations between cows and heifers with high and low fertility may allow researchers to manipulate the microbiota in cows with low fertility and increase their pregnancy rates, or use marker microbes as predictors of fertility in cows and heifers.

Determining methods to increase success of pregnancy and enhance fertility in domestic livestock would result in millions of dollars saved to producers annually. Providing foundational information and establishing baselines for native vaginal and uterine bacteriomes and complete microbiomes facilitates study of the impact of microbes on reproductive efficiency. In order to determine anomalies in the microbial communities that may lead to interference with successful pregnancies, healthy baselines should be established and shared throughout scientific and industrial communities. This study characterizes the native bacterial communities found in cows undergoing timed AI during a specific hormonal state. Future research may aim to determine the microbial communities during further stages of synchronization, or analyses to correlate the microbiome of the uterus and vagina with increased risk of disease, varying hormone levels throughout the reproductive cycle, changes introduced from sexual partners, or AI methods.

## Author Contributions

BC was responsible for primary manuscript preparation, sampling, and data and statistical analyses. SR, FD, GF, and OA were responsible for sampling and manuscript preparation. TS was responsible for DNA sequencing, sequencing analyses, and manuscript preparation. KP and PM were responsible for experimental design development, sampling, data and statistical analyses, and manuscript preparation.

## Conflict of Interest Statement

The authors declare that the research was conducted in the absence of any commercial or financial relationships that could be construed as a potential conflict of interest.
